# Age-specific association of stage of hypertension at diagnosis with cardiovascular and all-cause mortality among elderly patients with hypertension: a cohort study

**DOI:** 10.1186/s12872-023-03250-7

**Published:** 2023-05-23

**Authors:** Jingyi Wu, Xiaojie Han, Di Sun, Jie Zhang, Jiong Li, Guoyou Qin, Wei Deng, Yongfu Yu, Huilin Xu

**Affiliations:** 1 Department of Biostatistics, School of Public Health, The Key Laboratory of Public Health Safety of Ministry of Education, Fudan University, Shanghai, China; 2 The Fifth People’s Hospital of Shanghai, Fudan University, Shanghai, China; 3 Department of Cardiology, Shengli Oilfield Central Hospital, Dongying, Shandong China; 4 Department of Public Health, Aarhus University, Aarhus, Denmark; 5 NCRR-National Centre for Register-based Research, Aarhus University, Aarhus, Denmark; 6 Department of Clinical Medicine—Department of Clinical Epidemiology, Aarhus University, Aarhus, Denmark; 7 Shanghai Minhang Center for Disease Control and Prevention, Shanghai, China; 8 130 Dong’an Road, Shanghai, 200032 China; 9 965 Zhong Yi Road, Shanghai, 201101 China

**Keywords:** Hypertension, Age at diagnosis, Stage of hypertension, Cardiovascular mortality, Elderly

## Abstract

**Background:**

Hypertension affects 31.1% of adults worldwide, with higher prevalence of great than 60% in elderly. Advanced hypertension stage was associated with the higher risk of mortality. However, little is known about the age-specific association of stage of hypertension at diagnosis on cardiovascular mortality or all-cause mortality. Therefore, we aim to explore this age-specific association among the hypertensive elderly through stratified and interaction analyses.

**Methods:**

This cohort study included 125,978 elderly hypertensive patients aged 60+ years from Shanghai of China. Cox regression was used to estimate the independent and joint effect of hypertension stage and age at diagnosis on cardiovascular and all-cause mortality. Interactions were evaluated both additively and multiplicatively. Multiplicative interaction was examined by the Wald test of the interaction term. Additive interaction was assessed by relative excess risk due to interaction (RERI). All analyses were performed stratified by sex.

**Results:**

28,250 patients died during the follow-up up to 8.85 years, and 13,164 died of cardiovascular events. Older age and advanced hypertension stage were risk factors of cardiovascular mortality and all-cause mortality. Besides, smoking, rarely exercise, BMI < 18.5 and diabetes were also the risk factors. When we compared stage 3 hypertension with stage 1 hypertension, hazard ratios (95% confidence interval) of cardiovascular mortality and all-cause mortality were 1.56(1.41–1.72) and 1.29(1.21–1.37) for males aged 60–69 years, 1.25(1.14–1.36) and 1.13(1.06–1.20) for males aged 70–85 years, 1.48(1.32–1.67) and 1.29(1.19–1.40) for females aged 60–69 years, and 1.19(1.10–1.29) and 1.08(1.01–1.15) for females aged 70–85 years, respectively. Negative multiplicative interaction and positive additive interaction between age at diagnosis and stage of hypertension at diagnosis on cardiovascular mortality were observed in males (HR: 0.81, 95% CI: 0.71–0.93 RERI: 0.59, 95% CI: 0.09–1.07) and females (HR: 0.81, 95% CI: 0.70–0.93 RERI: 0.66, 95% CI: 0.10–1.23).

**Conclusions:**

Diagnosed with stage 3 hypertension was associated with higher risks of cardiovascular mortality and all-cause mortality, which were stronger among patients with age at diagnosis of 60–69 years compared with those with age at diagnosis of 70–85 years. Therefore, for the younger part of the elderly, the Department of Health should pay more attention to treating patients with stage 3 hypertension.

**Supplementary Information:**

The online version contains supplementary material available at 10.1186/s12872-023-03250-7.

## Introduction

Hypertension affected 31.1% of adults worldwide [1, 2]. In the elderly population, the prevalence even exceeded 60% [1]. The problem of aging is becoming increasingly severe and will continue throughout this century [3]. Thus, the number of elderly with hypertension will continuously increase, and the elderly with hypertension should be the point population of healthcare work.

Hypertension is the independent, direct and controllable risk factor for cardiovascular disease [1, 4–6], and cardiovascular disease is the leading cause of mortality around the world, especially in the low- and middle-income countries including China [7–9]. Also, cardiovascular disease is elderly’s leading cause of mortality [5, 10, 11], and because of the aging and growing older population, the global cardiovascular deaths is increasing [12, 13]. Therefore, the cardiovascular mortality as the important serious adverse event among the elderly with hypertension, was the interest outcome of this study. Hypertension can be classified as stage 1, stage 2 and stage 3 according to the level of elevated blood pressure, and the stage of hypertension is an important indicator for cardiovascular risk management in hypertensive patients [11]. Previous evidence has revealed the positive association between advanced stage of hypertension and increased risk of cardiovascular disease or cardiovascular mortality [5, 11, 14]. However, the evidence of the risk effect of advanced stage of hypertension on cardiovascular mortality in different age groups among the elderly hypertensive patients was still limited.

The elderly, the population with the heaviest disease burden, were always categorized as an entire subgroup. However, many studies have indicated that the younger elderly(< 70 years) and the older elderly(≥ 70 years) differed in many characteristics, such as physical or psychological health conditions, economic condition, relationship with society, and education level [15–18]. Today’s younger elderly lives in a younger life than before and we suspected that the age-specific association that the younger part of the elderly had higher risk of mortality exists in the elderly hypertensive patients. Therefore, it was necessary to explore the age-specific association of the stage of hypertension on cardiovascular mortality among the elderly and give suggestions for managing elderly hypertensive patients during the entire senior life course. And compared with the age and hypertension stage defined with cross-section data [14, 19–24], the age and the hypertension stage that were defined using the information of the first time they diagnosed with hypertension, may partly address the potential confounding effects caused by hypertension onset age [12] or potential misclassification of hypertension stage, which may cause by the treatment of hypertension.

In addition, limited previous research used interaction analysis to investigate the age-specific association between hypertension stage and mortality both in multiplicative and additive scales. Therefore, this current study aimed to explore the age-specific association between hypertension stage at diagnosis with cardiovascular mortality and all-cause mortality through stratified and interaction analyses among a hypertensive cohort, which enrolled patients once they were diagnosed with hypertension and recorded their age and hypertensive stage at that moment.

## Materials and methods

### Study population

The electronic health records (EHR) of Minhang District, Shanghai, China, is an electronic information system for the primary care management of hypertensive patients implemented in 2007. This EHR system collects the baseline and follow-up information of each patient.

**Fig. 1:**
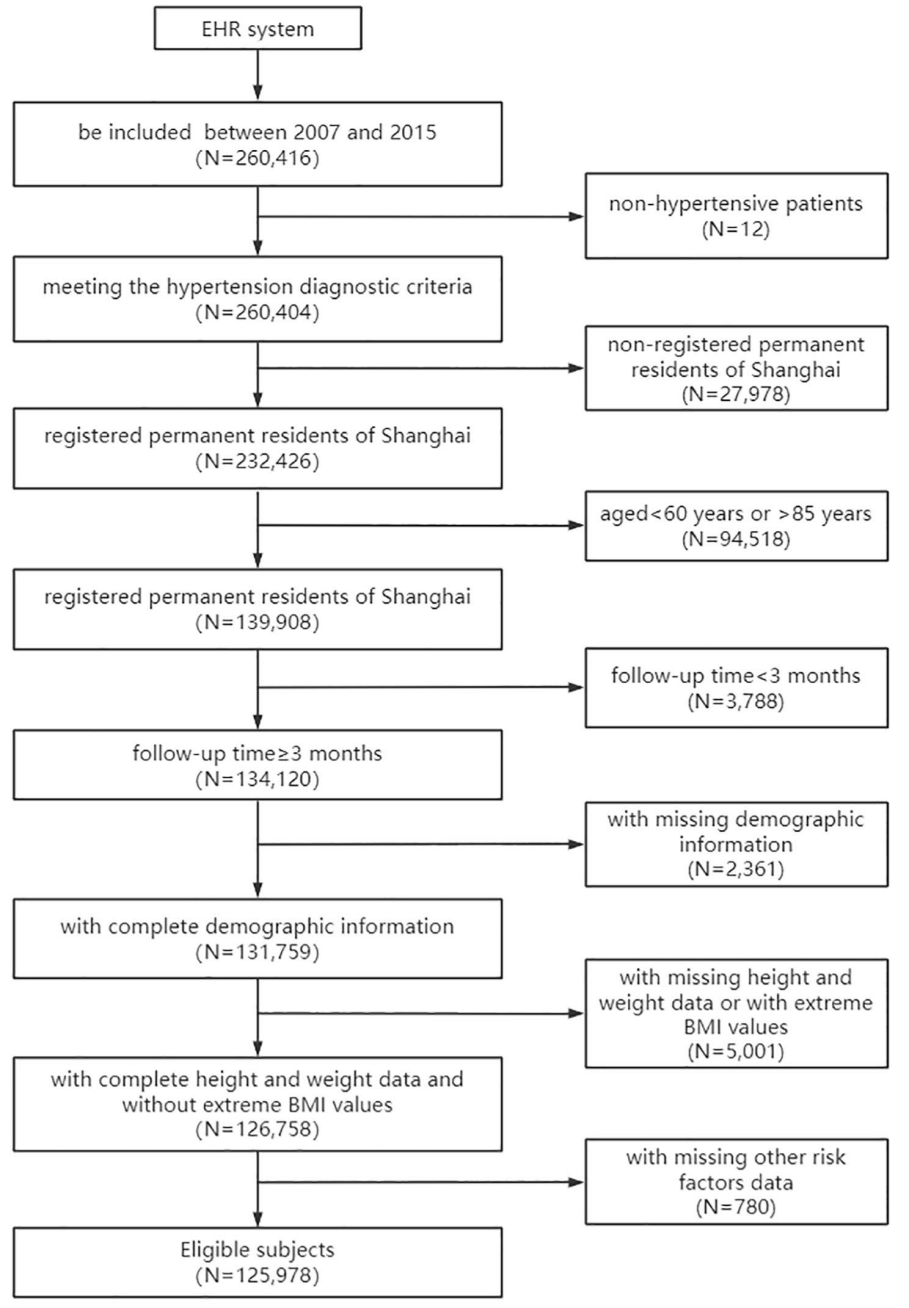
Flow chart of study sample inclusion

We acquired 260,416 patients who registered in the EHR system between 2007 and 2015 and were followed up until December 31, 2018. Finally, a total of 125,978 qualified subjects were included. The inclusion criteria were as follows: (1) meeting the hypertension diagnostic criteria defined by the World Health Organization (systolic blood pressure (SBP) ≥ 140mmHg or diastolic blood pressure (DBP) ≥ 90mmHg); (2) registered permanent residents of Shanghai; (3) aged 60–85 years old and (4) follow-up time ≥ 3 months. We excluded patients with missing demographic information, missing height, and weight data, or with extreme Body mass index(BMI) values and missing other risk factors data (Fig. 1).

### Exposure

The exposures of interest were age at diagnosis and the stage of hypertension at diagnosis. People were enrolled once they were diagnosed with hypertension through physical examination by the hospital or community. Therefore, we used the age enrolled in the EHR system(baseline age) to define the age at diagnosis, and it was divided into two groups (60–69 years and 70–85 years). Also, age at diagnosis would be treated as a continuous variable.

The three hypertension stages at diagnosis were defined according to the blood pressure at baseline(stage 1: SBP 140-159mmHg or DBP 90-99mmHg, stage 2: SBP 160-179mmHg or DBP 100-109mmHg, stage 3: SBP ≥ 180mmHg or DBP ≥ 110mmHg) [5, 11].

### Outcomes

The primary outcome was cardiovascular mortality, and the secondary outcome was all-cause mortality. All death information was confirmed by the death document provided by the Minhang District Center for Disease Control and Prevention.

### Covariates

The potential confounders in this study covered demographics, lifestyle, family history, and health status. Demographic variables included gender(Male/Female). Lifestyle variables were smoking, drinking, and physical exercise. Smoking was classified as yes(including quitting) and no, and “yes” was defined as smoking every day for a year; drinking was also classified as yes and no, and “yes” included as occasional drinking and regular drinking(consume alcohol at least twice a week for a year); physical exercise was classified as rarely exercise(nearly have no physical exercise), occasional exercise(< 150 min of moderate-intensity and < 75 min of high-intensity physical exercise per week) and regular exercise(≥ 150 min of moderate-intensity or ≥ 75 min of high-intensity physical exercise per week) [25, 26]. Family history variables contained family history of hypertension, coronary heart disease, stroke and diabetes (No/Yes). Comorbidities variables included diabetes in baseline(No/Yes) and BMI. If meeting the diagnostic criteria that fasting blood glucose ≥ 7.0 mmol/L or 2-hour blood glucose ≥ 11.1 mmol/L, diabetes comorbidities were diagnosed [27]. BMI was calculated as height/weight^2^, where the height and weight was self-reported and recorded in the EHR system. According to the previous studies and the presumed relationships between the covariates, exposures and outcomes(Supplementary Fig. 1), all these covariates would be included in the Cox model as confounders.

### Statistical analysis

The entire cohort was divided into six groups to describe the baseline characteristics according to the age group (60–69 years, 70–85 years) and stage of hypertension (stage 1, stage 2, stage 3). Continuous variables were described by median (interquartile range), and category variables were characterized by frequency (%). The Kruskal-Wallis test evaluated the difference between continuous variables, and the chi-square test assessed the difference between categorical variables.

We used generalized additive model to test for non-linear associations between continuous variables(age at diagnosis and BMI) and outcomes. We found a linear association between age at diagnosis and cardiovascular mortality as well as all-cause mortality and a non-linear association between BMI and cardiovascular mortality as well as all-cause mortality (Supplementary Fig. 2). Therefore, age at diagnosis would be analyzed as a categorical variable and continuous variable; BMI as a potential confounder would be included in the model as a categorical variable(BMI < 18.5/18.5 ≤ BMI < 25/25 ≤ BMI < 30/ BMI ≥ 30) [28].

The log-log survival (LLS) plot was used to evaluate whether the survival data satisfied the proportional hazard assumption of the Cox regression. In stratified analysis, Cox proportional hazards model was used to estimate the hazard ratio (HR) with 95% confidence intervals (95% CI) to assess the association between different stages of hypertension and cardiovascular mortality and all-cause mortality stratified by age groups and the association between age at diagnosis and cardiovascular mortality and all-cause mortality stratified by hypertension stages. In interaction analysis, the Cox proportional hazard models included age group, hypertension stage, and their interaction term. Multiplicative interaction was examined by the Wald test of the interaction term; additive interaction was measured by the relative excess risk of the interaction (RERI). Multiplicative and additive interactions have their own advantages: multiplicative interaction is easily to calculate in the Cox model and it is better suited for assessing causal associations; additive interaction is complicated to calculate but it is considered to have more biological or public health significance, which could indicate synergistic and antagonistic effects [23]. Thus, simultaneous estimation of multiplicative and additive interactions is encouraged. The potential confounding factors were included in the Cox proportion hazard model as covariates for adjustment except for the variable of gender, as the statistical analyses were stratified by it. An additional Cox model without any adjustment was also employed to support the stability of our results.

Considering the potential misclassification that patients registered in 2007 may have had outbreak hypertension before 2007, we excluded the patients registered in 2007, then conducted a sensitivity analysis to re-estimate the interaction between age and stage of hypertension at diagnosis.

All statistical tests in the current study were two-sided tests, and the P-value < 0.05 was considered statistically significant. All statistical analyses were performed using SAS version 9.4.

## Results

**Fig. 2:**
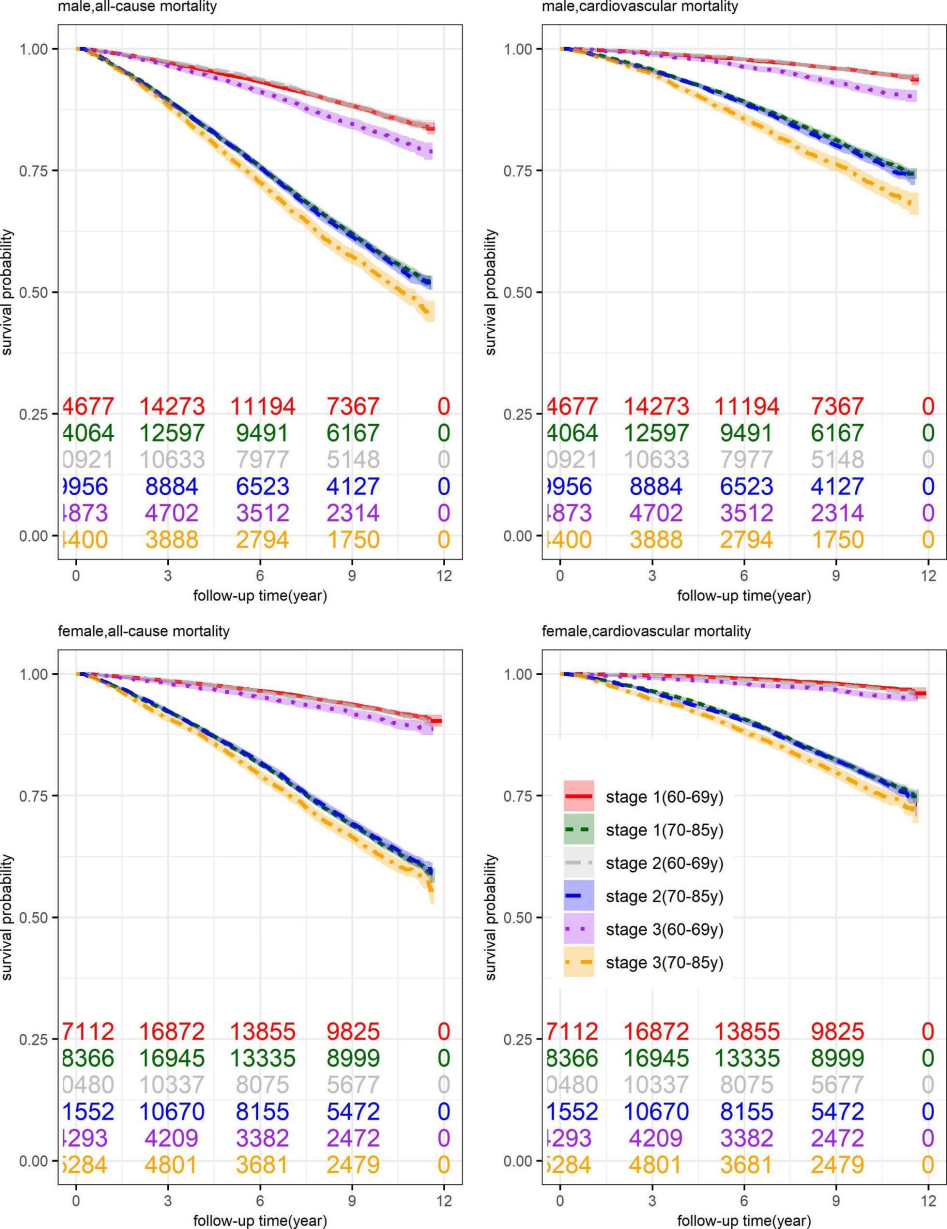
Age-specific survival curves and 95% CI band under different age groups and stages of hypertension

This study included 125,978 old hypertensive patients (60–85 years old) in Minhang District, Shanghai, with a median follow-up time of 8.85(4.82) years. A total of 28,250 patients died before December 31, 2018, of which 13,164 died of cardiovascular events. The patients who passed away were more likely to be older and diagnosed with advanced stage hypertension (Table 1). According to the survival curves (Fig. 2), we can intuitively observe that for patients more than 70 years old, their survival curves dropped rapidly. And patients diagnosed with stage 3 hypertension had lower survival curves than those diagnosed with stage 2 or 1 hypertension(the survival curves were similar between those diagnosed with stage 2 and stage 1 hypertension). Although older patients had a higher mortality risk, the number of new hypertensive patients decreased with age (Supplementary Fig. 3). The proportion of risk factors like smoking (20.60% versus 12.85%), drinking (20.65% versus 13.40%), family history of diabetes (5.47% versus 2.73%), coronary heart disease (2.93% versus 1.63%), hypertension (43.13% versus 25.27%), and stroke (2.76% versus 1.32%) were also higher among younger patients(60–69 years) (Table 1).

**Table 1:** Baseline characteristics by age and stage of hypertension

	Aged 60–69			Aged 70–85		
	Stage1 hypertension N = 31,789	Stage2 hypertension N = 21,401	Stage3 hypertension N = 9,166	Stage1 hypertension N = 32,430	Stage2 hypertension N = 21,508	Stage3 hypertension N = 9,684
**Man**(n%)	14,677(46.17)	10,921(51.03)	4873(53.16)	14,064(43.37)	9956(46.29)	4400(45.44)
**Age**	64.3(4.9)	64.2(5.0)	64.2(4.9)	75.9(6.5)	76.1(6.5)	76.1(6.6)
**Follow-up Person years**	9.7(4.2)	9.1(4.6)	9.5(4.6)	8.3(5.2)	7.7(5.3)	7.6(5.6)
**BMI**	23.9(3.7)	24.2(3.7)	24.4(4.0)	23.2(4.0)	23.4(3.9)	23.7(4.3)
**BMI < 18.5**	1119(2.47)	631(2.09)	301(2.29)	1079(5.72)	662(5.22)	306(5.36)
**18.5 ≤ BMI < 25**	29,443(64.93)	18,475(61.14)	7554(57.46)	13,146(69.66)	8536(67.26)	3630(63.64)
**25 ≤ BMI < 30**	13,358(29.46)	9905(32.78)	4635(35.26)	4202(22.27)	3110(24.51)	1563(27.40)
**BMI ≥ 30**	1427(3.15)	1207(3.99)	656(4.99)	445(2.36)	383(3.02)	205(3.59)
**Diabetes**(n%)	1476(4.64)	1268(5.92)	664(7.24)	756(2.33)	626(2.91)	358(3.70)
**Smoking**(n%)	5773(18.16)	4745(22.17)	2329(25.41)	3864(11.91)	2889(13.43)	1423(14.69)
**Drinking**(n%)	6104(19.20)	4683(21.88)	2091(22.81)	4189(12.92)	3020(14.04)	1319(13.62)
**Rarely exercise**(n%)	8577(26.98)	5688(26.58)	2529(27.59)	11,381(35.09)	7641(35.53)	3534(36.49)
**Occasional exercise**(n%)	14,744(46.38)	9937(46.43)	3915(42.71)	13,282(40.96)	8645(40.19)	3597(37.14)
**Regular Exercise**(n%)	8468(26.64)	5776(26.99)	2722(29.70)	7767(23.95)	5222(24.28)	2553(26.36)
**family history of diabetes**(n%)	1,892(4.2)	1,584(5.2)	829(6.3)	340(1.8)	310(2.4)	193(3.4)
**family history of stroke**(n%)	652(2.05)	639(2.99)	431(4.70)	324(1.00)	288(1.34)	229(2.36)
**family history of CVD**(n%)	678(2.13)	713(3.33)	435(4.75)	369(1.14)	358(1.66)	307(3.17)
**family history of HBP**(n%)	12,279(38.63)	9840(45.98)	4776(52.11)	7337(22.62)	5630(26.18)	3110(32.11)
**All-cause mortality**(n%)	2759(8.68)	1838(8.59)	1064(11.61)	11,419(35.21)	7444(34.61)	3726(38.48)
**CVD mortality**(n%)	916(2.88)	637(2.98)	457(4.99)	5506(16.98)	3685(17.13)	1963(20.27)

BMI: body mass index; HBP: high blood pressure; CVD: cardiovascular disease; Continuous variables were described by median (interquartile range); Category variables were described by frequency (%)

Statistical analyses showed no significant differences between stage 2 hypertension and stage 1 hypertension at diagnosis. Therefore, the differences talking about next were between stage 3 and stage 1 hypertension by default.

**Fig. 3:**
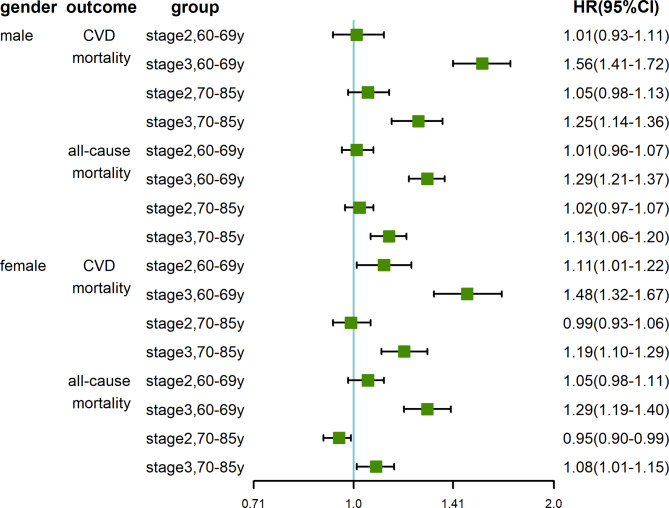
Forest plot of the age-specific effect of the stage of hypertension on the log scale Stage 1 hypertension as reference group

In the stratified analysis, when stratified by age groups, we observed a higher HR of stage 3 hypertension on cardiovascular mortality in the males aged 60–69 years(HR: 1.56, 95% CI: 1.41–1.72) than the males aged 70–85 years(1.25, 1.14–1.36) as well as all-cause mortality (1.29, 1.21–1.37 versus 1.13, 1.06–1.20), and we also observed this age-specific association in females. When stratified by hypertension stages, we found patients aged 70–85 had a significantly higher cardiovascular and all-cause mortality risk than patients aged 60–69 both in males(stage 1: 4.52, 4.19–4.87; stage 2: 4.55, 4.16–4.98; stage 3: 3.53, 3.14–3.97) and females(stage 1: 5.97, 5.54–6.43; stage 2: 5.27, 4.80–5.79; stage 3: 4.63, 4.09–5.25). Additionally, each year of age would also increase the risk of cardiovascular and all-cause mortality, and this association was attenuated as hypertension stage rose both in males(stage 1: 1.15, 1.14–1.16; stage 2: 1.15, 1.14–1.15; stage 3: 1.12, 1.11–1.13) and females(stage 1: 1.18, 1.17–1.19; stage 2: 1.17, 1.16–1.18; stage 3: 1.15, 1.14–1.16). (Table 2; Fig. 3) The results of males and females were similar, but we could notice that the increased age had a stronger impact on females and the advanced hypertension stage had a stronger impact on males.

**Table 2:** Stratified analysis of stage of hypertension or age on cardiovascular mortality and all-cause mortality

Hypertension stage	Age	N	Cardiovascular mortality			All-cause mortality		
			Mortality rate (per 10^3^)	HR ^a^	HR ^b^	Mortality rate (per 10^3^)	HR ^a^	HR ^b^
Man, stratified by age groups								
**Stage1**	60–69	14,677	557(4.64)	1(reference)	1(reference)	1665(13.87)	1(reference)	1(reference)
**Stage2**		10,921	395(4.54)	0.99(0.87–1.12)	1.01(0.93–1.11)	1167(13.4)	0.97(0.90–1.05)	1.01(0.96–1.07)
**Stage3**		4,873	306(7.87)	1.71(1.49–1.97)	1.56(1.41–1.72)	710(18.27)	1.33(1.21–1.45)	1.29(1.21–1.37)
**Stage1**	70–85	14,064	2411(22.82)	1(reference)	1(reference)	5502(52.09)	1(reference)	1(reference)
**Stage2**		9,956	1727(23.57)	1.04(0.98–1.11)	1.05(0.98–1.13)	3855(52.61)	1.02(0.97–1.06)	1.02(0.97–1.07)
**Stage3**		4,400	937(29.44)	1.31(1.21–1.41)	1.25(1.14–1.36)	1910(60.02)	1.16(1.10–1.23)	1.13(1.06–1.20)
Man, stratified by hypertension stages: age 60–69 as reference group								
**Stage1**	70–85	14,064	2411(22.82)	4.99(4.55–5.47)	4.52(4.19–4.87)	5502(52.09)	3.80(3.60–4.01)	3.56(3.40–3.74)
**Stage2**	70–85	9,956	1727(23.57)	5.26(4.71–5.86)	4.55(4.16–4.98)	3855(52.61)	3.97(3.72–4.24)	3.61(3.41–3.82)
**Stage3**	70–85	4,400	937(29.44)	3.82(3.35–4.34)	3.53(3.14–3.97)	1910(60.02)	3.34(3.06–3.64)	3.13(2.89–3.39)
**Stage1**	age(continuous)	28,741	2968(13.15)	1.15(1.14–1.16)	1.15(1.14–1.16)	7167(31.76)	1.12(1.12–1.13)	1.12(1.12–1.13)
**Stage2**		20,877	2122(13.23)	1.15(1.14–1.16)	1.15(1.14–1.15)	5022(31.32)	1.13(1.12–1.13)	1.12(1.12–1.13)
**Stage3**		9273	1243(17.59)	1.12(1.11–1.13)	1.12(1.11–1.13)	2620(37.07)	1.11(1.10–1.12)	1.11(1.10–1.11)
Women, stratified by age groups								
**Stage1**	60–69	17,112	359(2.44)	1(reference)	1(reference)	1094(7.44)	1(reference)	1(reference)
**Stage2**		10,480	242(2.75)	1.14(0.97–1.34)	1.11(1.01–1.22)	671(7.64)	1.04(0.94–1.14)	1.05(0.98–1.11)
**Stage3**		4,293	151(4.12)	1.68(1.39–2.04)	1.48(1.32–1.67)	354(9.65)	1.30(1.15–1.46)	1.29(1.19–1.40)
**Stage1**	70–85	18,366	3095(21.28)	1(reference)	1(reference)	5917(40.68)	1(reference)	1(reference)
**Stage2**		11,552	1958(21.72)	1.03(0.97–1.09)	0.99(0.93–1.06)	3589(39.81)	0.98(0.94–1.02)	0.95(0.90–0.99)
**Stage3**		5,284	1026(25.11)	1.19(1.11–1.27)	1.19(1.10–1.29)	1816(44.44)	1.10(1.04–1.16)	1.08(1.01–1.15)
Women, stratified by hypertension stages: age 60–69 as reference group								
**Stage1**	70–85	18,366	3095(21.28)	8.91(7.99–9.94)	5.97(5.54–6.43)	5917(40.68)	5.56(5.21–5.93)	4.39(4.17–4.61)
**Stage2**	70–85	11,552	1958(21.72)	8.02(7.02–9.17)	5.27(4.80–5.79)	3589(39.81)	5.28(4.86–5.73)	3.94(3.70–4.21)
**Stage3**	70–85	5,284	1026(25.11)	6.23(5.25–7.39)	4.63(4.09–5.25)	1816(44.44)	4.69(4.18–5.25)	3.67(3.36–4.01)
**Stage1**	age(continuous)	35,478	3454(11.81)	1.19(1.18–1.19)	1.18(1.17–1.19)	7011(23.96)	1.15(1.14–1.15)	1.14(1.14–1.15)
**Stage2**		22,032	2200(12.36)	1.18(1.17–1.18)	1.17(1.16–1.18)	4260(23.93)	1.14(1.14–1.15)	1.14(1.13–1.14)
**Stage3**		9577	1177(15.18)	1.16(1.15–1.17)	1.15(1.14–1.16)	2170(27.98)	1.13(1.13–1.14)	1.13(1.12–1.14)

Mortality rate: mortality number / person-years

^a^ Unadjusted; ^b^ Adjusted for smoke, drink, diabetes, body mass index, family history of diabetes, family history of stroke, family history of cardiovascular disease, family history of hypertension

Besides, we estimated the HR(95% CI) of confounders in our study. The results showed that smoking, rarely exercise, BMI < 18.5 and diabetes were the risk factors of cardiovascular mortality and all-cause mortality. However, the hazard effect of smoking was nonsignificant among females, which might because only 1.11% of women had the habit of smoking. And the results showed that drinking and having family history of high blood pressure and diabetes were protected factors of mortality.(Supplementary Table 1).

Next, there was a negative multiplicative interaction between age and stage 3 hypertension on cardiovascular and all-cause mortality in males(cardiovascular mortality: HR: 0.81, 95% CI: 0.71–0.93; all-cause mortality: HR: 0.90, 95% CI: 0.82–0.98) and females(cardiovascular mortality: HR: 0.81, 95% CI: 0.70–0.93; all-cause mortality: HR: 0.85, 95% CI: 0.77–0.93), and stronger associations were observed in cardiovascular mortality (Table 3). Additionally, we observed positive additive interaction between age and stage 3 hypertension on cardiovascular mortality in males(RERI: 0.58, 95% CI: 0.09–1.07) and females(RERI: 0.66, 95% CI: 0.10–1.23) and this positive additive interaction was significantly stronger in females.

**Table 3:** Interaction of age and stage of hypertension on cardiovascular mortality and all-cause mortality

Age	hypertension stage	cardiovascular mortality		all-cause mortality		
		HR ^a^	HR ^b^	HR ^a^	HR ^b^	
male						
60–69	stage 1	1(reference)	1(reference)	1(reference)	1(reference)	
60–69	stage 2	1.01(0.92–1.10)	1.01(0.88–1.14)	0.98(0.93–1.04)	1.00(0.95–1.06)	
60–69	stage 3	1.55(1.40–1.71)	1.75(1.52–2.01)	1.25(1.17–1.33)	1.28(1.20–1.36)	
70–85	stage 1	4.43(4.11–4.77)	4.80(4.37–5.26)	3.72(3.55–3.89)	3.54(3.38–3.72)	
70–85	stage 2	4.67(4.31–5.06)	5.05(4.59–5.56)	3.77(3.58–3.97)	3.63(3.44–3.82)	
70–85	stage 3	5.56(5.06–6.11)	6.39(5.75–7.10)	4.19(3.93–4.46)	4.06(3.81–4.33)	
female						
60–69	stage 1	1(reference)	1(reference)	1(reference)	1(reference)	
60–69	stage 2	1.09(0.98–1.20)	1.10(1.00-1.22)	1.03(0.96–1.09)	1.04(0.98–1.11)	
60–69	stage 3	1.44(1.28–1.62)	1.48(1.31–1.66)	1.26(1.17–1.36)	1.28(1.19–1.39)	
70–85	stage 1	6.47(6.02–6.96)	5.89(5.47–6.34)	4.74(4.52–4.98)	4.36(4.15–4.58)	
70–85	stage 2	6.42(5.94–6.95)	5.87(5.42–6.35)	4.50(4.26–4.75)	4.14(3.92–4.37)	
70–85	stage 3	7.56(6.89–8.29)	7.03(6.41–7.72)	5.08(4.75–5.43)	4.73(4.43–5.06)	
**Interaction on multiplicative scale**		**HR** ^**a**^	**HR** ^**b**^	**HR** ^**a**^	**HR** ^**b**^	
male	70–85*stage 2	1.06(0.94–1.18)	1.05(0.93–1.17)	1.03(0.96–1.11)	1.02(0.95–1.10)	
	70–85*stage 3	0.82(0.72–0.93)	0.81(0.71–0.93)	0.91(0.83–0.99)	0.90(0.82–0.98)	
female	70–85*stage 2	0.91(0.81–1.03)	0.90(0.80–1.02)	0.92(0.85-1.00)	0.91(0.84–0.99)	
	70–85*stage 3	0.81(0.70–0.94)	0.81(0.70–0.93)	0.85(0.77–0.94)	0.85(0.77–0.93)	
**Interaction on additive scale**		**RERI** ^**a**^	**RERI** ^**b**^	**RERI** ^**a**^	**RERI** ^**b**^	
male	70–85*stage 2	0.23(-0.12-0.59)	0.23(-0.11-0.57)	0.07(-0.12-0.26)	0.08(-0.11-0.26)	
	70–85*stage 3	0.61(0.10–1.12)	0.58(0.09–1.07)	0.23(-0.04-0.49)	0.24(-0.02-0.50)	
female	70–85*stage 2	-0.13(-0.56-0.29)	-0.13(-0.52-0.26)	-0.27(-0.50–0.04)	-0.26(-0.48–0.05)	
	70–85*stage 3	0.65(0.04–1.25)	0.66(0.10–1.23)	0.07(-0.25-0.39)	0.09(-0.21-0.39)	

^a^ Unadjusted; ^b^ Adjusted for smoke, drink, diabetes, body mass index, family history of diabetes, family history of stroke, family history of cardiovascular disease, family history of hypertension

In sensitivity analyses, patients registered in 2007 were excluded for reduced probability of misclassification. The results were consistent with the previous one and even more significant. For example, we observed the negative multiplicative interaction (HR: 0.77, 95% CI: 0.64–0.92) and positive additive interaction (RERI: 0.95, 95% CI: 0.36–1.53) in males on cardiovascular mortality (Supplementary Table 2).

## Discussion

In this large population-based cohort study, advanced stage of hypertension and older age at diagnosis were independently associated with a higher risk of cardiovascular and all-cause mortality. And the association of stage 3 hypertension and mortality was stronger in the elderly aged 60–69 years. It was the first study to identify the negative multiplicative and positive additive interaction between hypertension stage and age at diagnosis on mortality. The negative multiplicative interaction indicated that the relative risk(ratio) of cardiovascular and all-cause mortality due to stage 3 hypertension decreased with aging. The positive additive interaction implied that the absolute risk(difference) of cardiovascular mortality increased with aging.

Hypertension is a chronic disease generally occurring in the elderly [1, 2, 4], because aging reduces vascular resistance and cardiac output, eventually leading to hypertension [10]. Therefore, we chose elderly hypertensive patients as target research subjects. The adverse effect of advanced stage hypertension on cardiovascular mortality has also been widely acknowledged, but this study highlighted the hypertension stage at diagnosis. On the one hand, using the hypertension stage at diagnosis can avoid misclassification in cross-sectional studies, which may be due to antihypertensive drugs or other treatments. On the other hand, taking the stage of hypertension at diagnosis as the exposure can be helpful to achieve appropriate management and treatment from the onset of hypertension diagnosis. The results of this study suggested patients diagnosed with stage 3 hypertension need strong attention, and patients diagnosed with stage 2 hypertension may be treated in the same way as stage 1 hypertension. Focus on the effect of confounders, we found drinking was a protected factor of mortality. According to other studies, light drinking is protective against mortality [29, 30]. We cannot access the information of alcohol consumption, but it was reasonable to hypothesize that the drinkers in our study tends to consume lower levels of alcohol. Besides, having family history of high blood pressure and diabetes were also protected factors of mortality. One possible explanation is that this cohort is a hypertension cohort, and those who have a family history of chronic diseases may be more knowledgeable in controlling their disease condition, leading to a reduced risk of mortality in the hypertension cohort.

For the age-specific association of hypertension stage with mortality, we found the negative multiplicative interaction between age and stage 3 hypertension among the elderly hypertensive patients, which is similar with the previous studies’ results. Some previous studies had demonstrated that the younger’s relative risk of cardiovascular diseases caused by hypertension was significantly higher than the elder’s among the whole adults(≥ 20 years) [14, 19–23, 31–33]. And there were generally two potential underlying mechanisms to explain this age-specific association. One was the genetic mechanism: some studies speculated that some genetic loci and gene variants were associated with early-onset hypertension, and some of them were also associated with cardiovascular disease; [34–36] the other potential underlying mechanism was about organ damage: in the CARDIA study, Karri, et al. excluded the effect of the course of the disease and found the association of early onset of hypertension with hypertension mediated organ damage, which showed that the young people had the higher risk of organ damage and then may cause the higher risk of cardiovascular events [22]. However, among the elderly, these two mechanisms seemed lack of persuasiveness, because the new-onset hypertension of the elderly were not belong to the early-onset hypertension. Another potential mechanism may better explain the negative multiplicative interaction between age and stage 3 hypertension among the elderly hypertensive patients. Some studies indicated that in the oldest people, the blood pressure showed a “J” or “U” shape association with mortality [24, 37, 38]. Dalen, et al. observed that the systolic blood pressure associated with the lowest risk of mortality was 134 mmHg in the elderly aged 60–70 and was over 160 mmHg in the elderly over 70 years old [38]. And Ogliari, et al. found the systolic blood pressure of 165 mmHg and the diastolic blood pressure of 85 mmHg were associated with the lowest risk of mortality in the cohort of elderly older than 75 years old [37]. We suspected that this “J” or “U” shape association in the oldest elderly may lead to the lower HR of advanced hypertension stage compared with stage 1 hypertension among elderly aged 70–85. Anyway, the relevant mechanisms of negative multiplicative interaction between age and stage 3 hypertension among the elderly hypertensive population are lacking and more research are required to discover it.

We further identified a positive additive interaction between age and stage 3 hypertension, which is more of public health significance when compared with the negative multiplicative interaction [39]. The observed positive additive interaction meant the elderly aged 70–85 with stage 3 hypertension was the population of highest absolute cardiovascular mortality risk and the treatment of hypertension on them would avoid more absolute cardiovascular mortality burden among hypertensive patients. A participant-level meta-analyses showed a similar conclusion with this study, which found that the floating absolute risks of SBP or DBP on cardiovascular mortality as well as all-cause mortality will increase with aging [20]. Anyway, limited literatures described additive interactions of age groups and blood pressure to measure the absolute mortality risk, but simply put, the rate of mortality can be another indicator of absolute risk of mortality [40]. And we all know that age is an extremely important factor of mortality, so the elderly aged 70–85 with stage 3 hypertension was the population of highest absolute cardiovascular mortality risk was reasonable. In addition, the positive additive interaction was significantly stronger in females than in male. We suspected this difference between males and females was due to the effect of sex hormones. The increase in the androgen ratio in older women after menopause, estradiol, anxiety, and other factors will become a new but essential mechanism of hypertension for elderly females [41]. Some articles pointed out that compared with men, the non-dipping blood pressure pattern in females after menopause will lead to worse cardiovascular status [42]. However, almost women aged 60 to 69 have also been postmenopausal. Studying the relationship between the duration of menopause and hypertension or cardiovascular disease may be helpful. Other literature found that the difference between males and females in cardiovascular diseases may be directly affected by X chromosome [43]. However, this is now enough to enlighten us that we need to pay more attention to elderly hypertensive females.

This current study has some advantages. Firstly, we conducted this research in a large prospective community hypertensive cohort, which recorded the age and hypertension stage at diagnosis. For that, we can avoid some potential confounding effects or misclassification as described in the introduction. Secondly, we simultaneously estimated the multiplicative and additive interaction between age and hypertension stage. To our knowledge, multiplicative interactions were suitable for reflecting relative risk, while the additive interaction reflects the absolute risk, which can better indicate the public health significance [39]. Combing two types of interaction will bring complete information about the association of hypertension stage and age between mortality. Thirdly, our data had sufficient covariates information, including lifestyle, family history, etc.

There are also some limitations to this study. Firstly, we can sometimes not obtain the exact age at diagnosis because of delayed reporting and other conditions. But this system had been developed for information collection, recording, and transmission of standard operating procedures and had full-time supervisors for routine quality control [44], so this situation should be within the acceptance range. Besides, this current study performed a sensitivity analysis that excluded the people who registered in 2007, the year most likely occurred delayed reporting, and the consistent result strengthened the reliability of this study. Secondly, secondary hypertension was not taken into account in this study. However, previous studies have found that secondary hypertension accounts for only 5–10% of patients with hypertension [44], with less impact on outcomes. Thirdly, the European Guidelines for the Treatment of Arterial Hypertension suggested that systolic pressure of less than 120mmHg and a diastolic pressure of less than 80mmHg are ideal blood pressure [2, 45], but this study did not include the population whose blood pressure was over the ideal blood pressure but did not meet the hypertension criteria. Fourthly, the popularity of this study is limited. Our research population was only from the Shanghai Minhang District, not considering other countries, regions, and races. Further studies are needed to provide scientific evidence to fill those vacancies. Finally, our study has limitations in terms of the available data on comorbidities, with only diabetes being included as a comorbidity in our dataset. However, lifestyle factors can be proxy variables for some comorbidities. For example, smoking can be a potential proxy for COPD for almost 90% COPD(chronic obstructive pulmonary disease) were caused by it [46, 47]. Besides, a meta-analysis showed that physical exercise, drinking and smoking were risk factors of kidney diseases [48]. Therefore, through adjusted lifestyle factors in the model, the potential confounding effect of unobserved comorbidities may be mitigated.

## Conclusions

Diagnosed with stage 3 hypertension was associated with higher cardiovascular and all-cause mortality risk when compared with stage 1 hypertension among the elderly hypertensive patients. And this current study highlighted that in the younger part of the elderly, this association was stronger than that in the elderly part of elderly. Thus, for the younger part of the elderly, the Department of Health should pay more attention to treat the patients with stage 3 hypertension. This current study also pointed out that the older elderly has a huge absolute mortality risk, and the mortality risk under stage 1 hypertension was already very high, so for the older part of the elderly, patients with all the stage of hypertension need pay great attention to.

## Electronic supplementary material

Below is the link to the electronic supplementary material.Supplementary Figure 1. Directed acyclic graph. Supplementary Figure 2. Exposure response curves for age at diagnosis and body mass index(BMI) in the generalized additive model. Supplementary Figure 3. Age-specific probability density distribution chart of age at diagnosis. Supplementary Table 1. Stratified analysis of confounders on cardiovascular mortality and all-cause mortality. Supplementary Table 2. Interaction of age at diagnosis and stage of hypertension on cardiovascular mortality and all-cause mortality when registered after 2007.

Additional File:

## Data Availability

The datasets used and analysed during the current study are available from the corresponding author on reasonable request. The dataset supporting the conclusions of this article is included within the article and its additional file1.
