# Supplementary material for: Age-specific association of stage of hypertension at diagnosis with cardiovascular and all-cause mortality among elderly patients with hypertension: a cohort study
Source: BMC Cardiovasc Disord. 2023 May 23;23:270. doi: 10.1186/s12872-023-03250-7 (PMC10207764; doi:10.1186/s12872-023-03250-7)
Supplement: Supplementary file 1 — Additional File: [file 12872_2023_3250_MOESM1_ESM.doc]

**Supplementary Material**

**Supplementary Figure 1.** **Directed acyclic graph.**


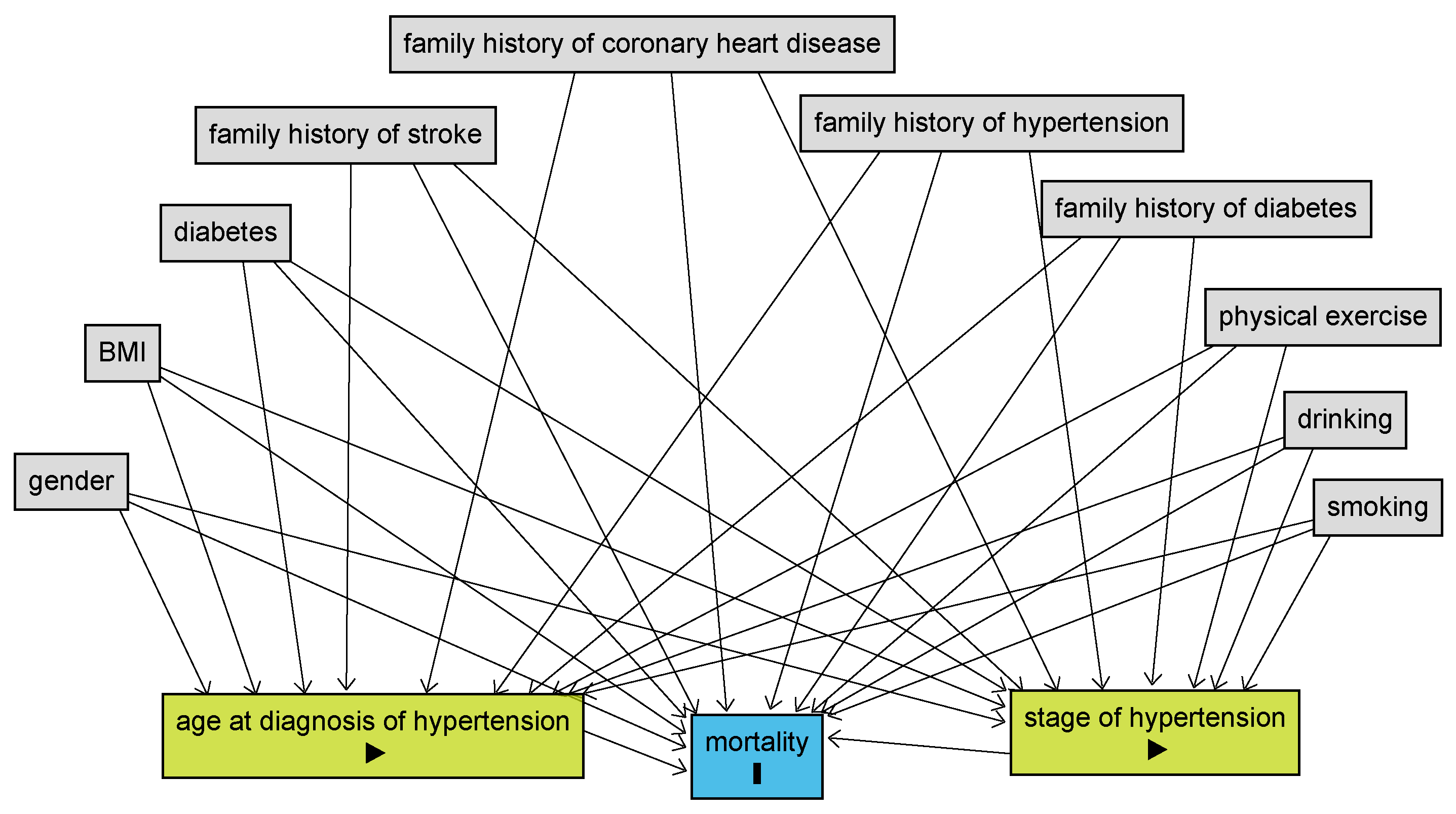


Yellow represents exposure;

Blue represents outcome;

Gray represents confounding factors.

**Supplementary Figure 2.** **Exposure response curves for age at diagnosis and body mass index(BMI) in the generalized additive model.**


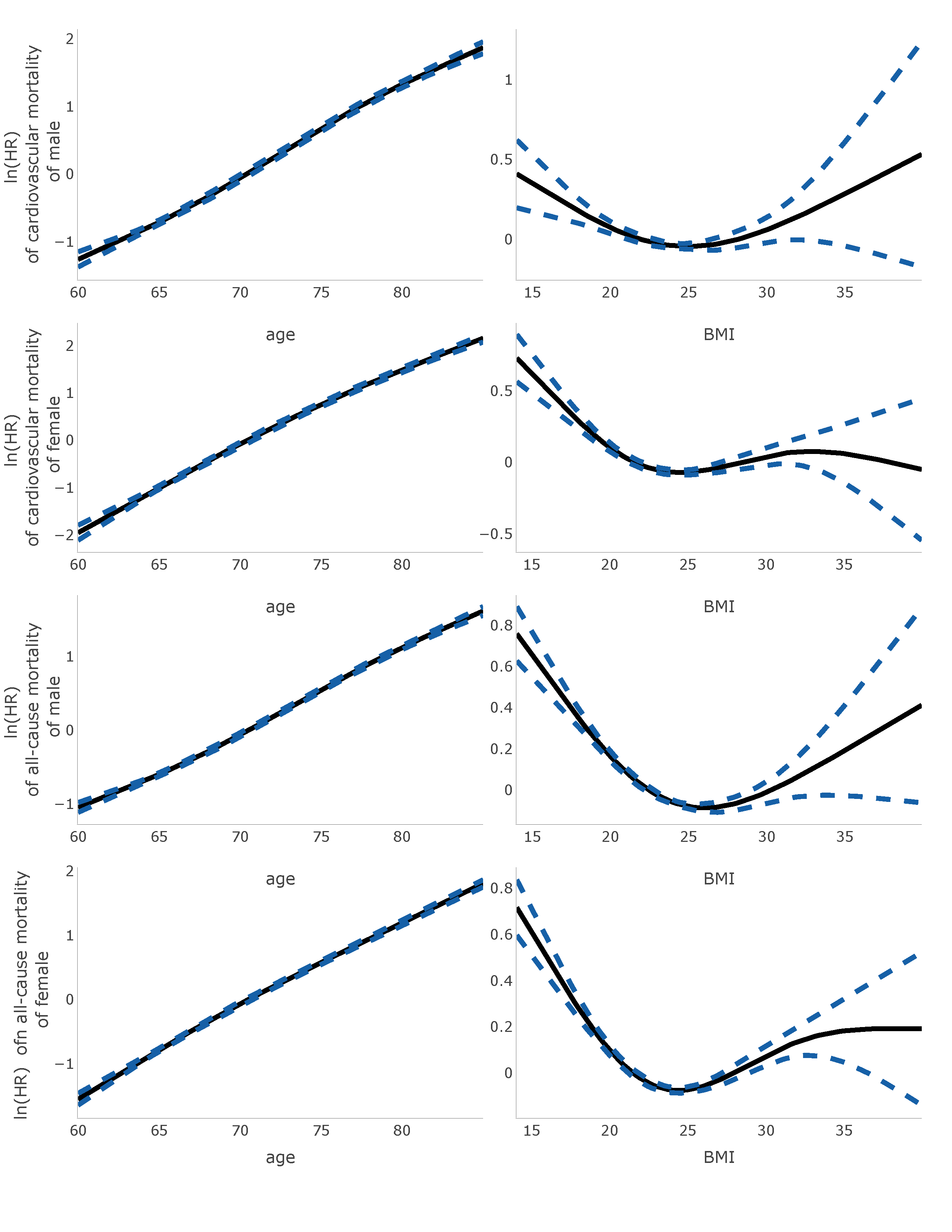


**Supplementary Figure 3. Age-specific probability density distribution chart of age at diagnosis.**


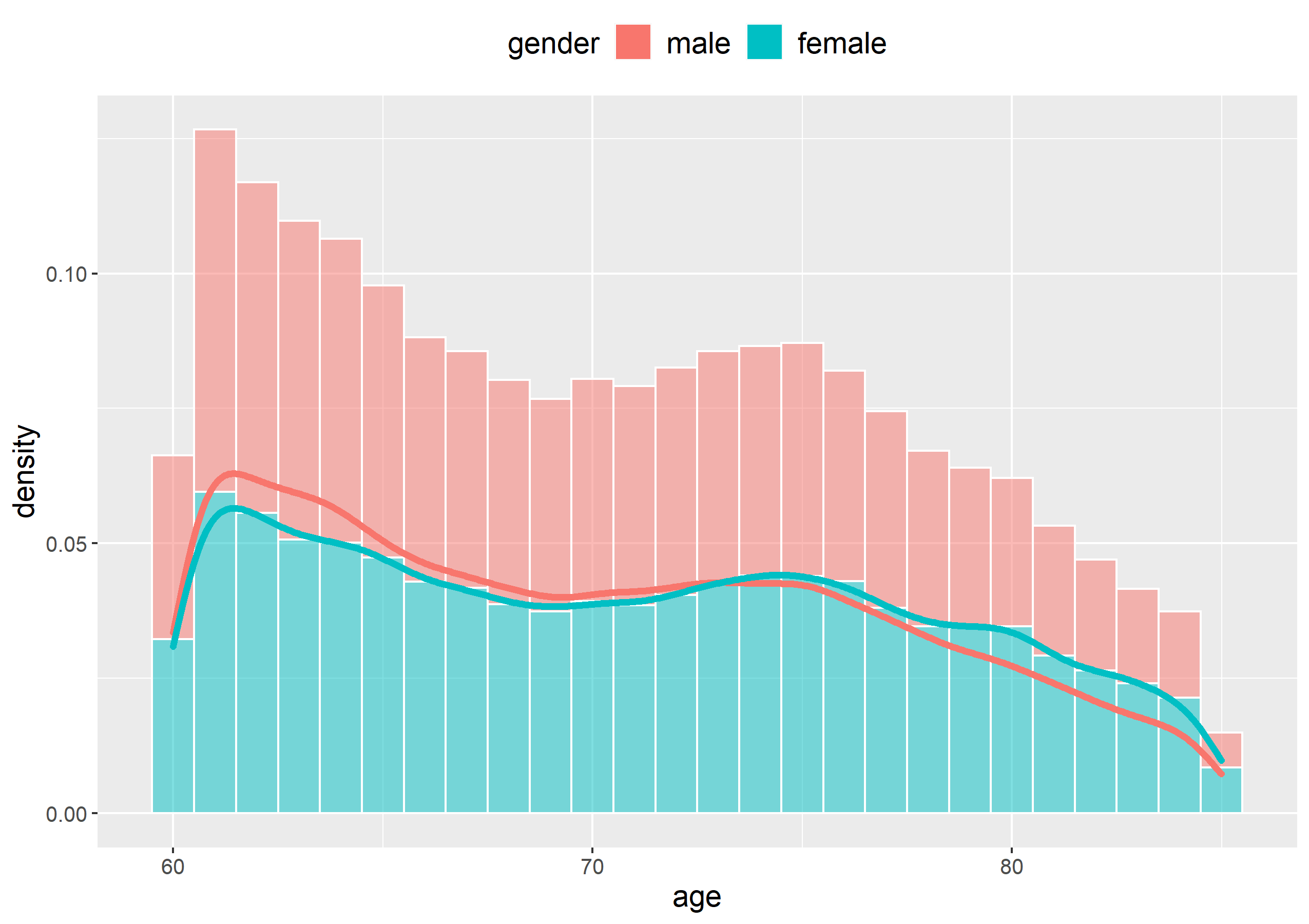


**Supplementary Table 1. Stratified analysis of confounders on cardiovascular mortality and all-cause mortality**

|  | **Cardiovascular mortality** | | **All-cause mortality** | |
| --- | --- | --- | --- | --- |
| **Confounders** | 60-69 years old | 70-85 years old | 60-69 years old | 70-85 years old |
| **male** |  |  |  |  |
| smoking | 1.26(1.12-1.42) | 1.11(1.04-1.18) | 1.33(1.24-1.43) | 1.22(1.17-1.27) |
| drinking | 0.72(0.64-0.82) | 0.81(0.76-0.87) | 0.83(0.77-0.90) | 0.84(0.81-0.88) |
| diabetes | 1.65(1.46-1.88) | 1.15(1.07-1.23) | 1.59(1.47-1.71) | 1.28(1.22-1.34) |
| rarely exercise | 1(reference) |  |  |  |
| occasional exercise | 0.69(0.61-0.78) | 0.62(0.58-0.66) | 0.76(0.70-0.82) | 0.70(0.67-0.73) |
| regular exercise | 0.57(0.49-0.65) | 0.48(0.44-0.51) | 0.67(0.62-0.73) | 0.54(0.52-0.57) |
| BMI<18.5 | 2.12(1.58-2.85) | 1.39(1.23-1.58) | 2.15(1.81-2.57) | 1.55(1.43-1.68) |
| 18.5≤BMI<25 | 1(reference) |  |  |  |
| 25≤BMI<30 | 0.85(0.75-0.96) | 0.94(0.88-1.01) | 0.83(0.77-0.89) | 0.87(0.83-0.91) |
| BMI≥30 | 1.00(0.73-1.38) | 1.05(0.86-1.27) | 0.96(0.79-1.16) | 0.91(0.79-1.05) |
| Family history of HBP | 0.96(0.85-1.08) | 0.88(0.83-0.95) | 0.89(0.83-0.96) | 0.86(0.82-0.90) |
| Family history of CVD | 0.93(0.65-1.33) | 1.08(0.87-1.36) | 0.78(0.62-0.99) | 0.92(0.78-1.08) |
| Family history of stroke | 1.13(0.81-1.58) | 0.94(0.74-1.21) | 0.94(0.75-1.18) | 0.88(0.74-1.05) |
| Family history of diabetes | 1.07(0.84-1.35) | 0.73(0.59-0.90) | 1.02(0.87-1.18) | 0.84(0.74-0.96) |
| **female** |  |  |  |  |
| smoking | 1.17(0.55-2.50) | 1.17(0.94-1.45) | 1.26(0.81-1.95) | 1.22(1.05-1.43) |
| drinking | 0.95(0.55-1.62) | 0.99(0.82-1.18) | 0.92(0.67-1.26) | 0.95(0.83-1.08) |
| diabetes | 2.08(1.79-2.43) | 1.27(1.20-1.35) | 1.81(1.65-1.99) | 1.42(1.36-1.48) |
| rarely exercise | 1(reference) |  |  |  |
| occasional exercise | 0.68(0.58-0.81) | 0.68(0.65-0.72) | 0.72(0.65-0.79) | 0.73(0.70-0.76) |
| regular exercise | 0.72(0.60-0.86) | 0.56(0.52-0.60) | 0.65(0.58-0.73) | 0.60(0.57-0.64) |
| BMI<18.5 | 2.20(1.54-3.15) | 1.56(1.41-1.72) | 2.59(2.12-3.16) | 1.49(1.38-1.60) |
| 18.5≤BMI<25 | 1(reference) |  |  |  |
| 25≤BMI<30 | 0.96(0.82-1.12) | 0.89(0.84-0.95) | 0.99(0.90-1.09) | 0.91(0.87-0.95) |
| BMI≥30 | 0.84(0.59-1.20) | 0.97(0.85-1.11) | 1.14(0.94-1.38) | 1.08(0.98-1.18) |
| Family history of HBP | 0.76(0.65-0.89) | 0.82(0.77-0.88) | 0.81(0.74-0.89) | 0.85(0.82-0.89) |
| Family history of CVD | 0.81(0.50-1.31) | 1.06(0.86-1.30) | 0.92(0.70-1.21) | 0.98(0.84-1.14) |
| Family history of stroke | 0.92(0.55-1.53) | 1.11(0.88-1.41) | 0.71(0.51-1.00) | 0.99(0.82-1.18) |
| Family history of diabetes | 0.62(0.42-0.90) | 0.72(0.60-0.86) | 0.72(0.58-0.89) | 0.78(0.69-0.89) |

BMI: body mass index; HBP: high blood pressure; CVD: cardiovascular disease.

**Supplementary Table 2. Interaction of age at diagnosis and stage of hypertension on cardiovascular mortality and all-cause mortality when registered after 2007**

|  |  | **cardiovascular mortality** | | **all-cause mortality** | |
| --- | --- | --- | --- | --- | --- |
| **Age** | **hypertension stage** | **HR ^a^** | **HR ^b^** | **Age** | **hypertension stage** |
| male | | | | | |
| 60-69 | stage 1 | 1(reference) | 1(reference) | 1(reference) | 1(reference) |
| 60-69 | stage 2 | 0.98(0.85-1.13) | 1.00(0.86-1.15) | 0.97(0.90-1.06) | 0.99(0.91-1.08) |
| 60-69 | stage 3 | 1.72(1.48-2.02) | 1.76(1.50-2.05) | 1.33(1.21-1.47) | 1.36(1.24-1.50) |
| 70-85 | stage 1 | 5.12(4.62-5.68) | 4.88(4.40-5.41) | 3.85(3.62-4.09) | 3.68(3.46-3.91) |
| 70-85 | stage 2 | 5.53(4.97-6.14) | 5.28(4.75-5.88) | 3.96(3.72-4.22) | 3.80(3.57-4.05) |
| 70-85 | stage 3 | 6.80(6.04-7.64) | 6.57(5.84-7.39) | 4.47(4.15-4.81) | 4.36(4.05-4.69) |
| female | | | | | |
| 60-69 | stage 1 | 1(reference) | 1(reference) | 1(reference) | 1(reference) |
| 60-69 | stage 2 | 1.13(0.94-1.36) | 1.16(0.96-1.39) | 1.01(0.91-1.12) | 1.02(0.92-1.14) |
| 60-69 | stage 3 | 1.86(1.51-2.29) | 1.93(1.57-2.38) | 1.34(1.17-1.53) | 1.38(1.21-1.58) |
| 70-85 | stage 1 | 9.21(8.15-10.40) | 8.33(7.36-9.41) | 5.66(5.27-6.08) | 5.17(4.81-5.55) |
| 70-85 | stage 2 | 9.37(8.26-10.63) | 8.54(7.53-9.69) | 5.48(5.09-5.91) | 5.03(4.66-5.42) |
| 70-85 | stage 3 | 11.28(9.86-12.90) | 10.46(9.14-11.97) | 6.32(5.81-6.86) | 5.87(5.40-6.38) |
| **Interaction on multiplicative scale** | | **HR ^a^** | **HR ^b^** | **HR ^a^** | **HR ^b^** |
| male | 70-85*stage 2 | 1.10(0.94-1.29) | 1.09(0.93-1.27) | 1.06(0.96-1.16) | 1.04(0.95-1.15) |
|  | 70-85*stage 3 | 0.77(0.64-0.92) | 0.77(0.64-0.92) | 0.87(0.78-0.98) | 0.87(0.78-0.98) |
| female | 70-85*stage 2 | 0.90(0.74-1.09) | 0.89(0.73-1.08) | 0.96(0.86-1.08) | 0.95(0.85-1.07) |
|  | 70-85*stage 3 | 0.66(0.53-0.82) | 0.65(0.52-0.81) | 0.83(0.72-0.96) | 0.82(0.71-0.95) |
| **Interaction on additive scale** | | **RERI ^a^** | **RERI ^b^** | **RERI ^a^** | **RERI ^b^** |
| male | 70-85*stage 2 | 0.42(0.03-0.81) | 0.41(0.04-0.78) | 0.14(-0.06-0.33) | 0.14(-0.05-0.32) |
|  | 70-85*stage 3 | 0.95(0.36-1.53) | 0.94(0.37-1.50) | 0.29(0.01-0.56) | 0.32(0.05-0.60) |
| female | 70-85*stage 2 | 0.04(-0.59-0.66) | 0.06(-0.51-0.63) | -0.19(-0.47-0.09) | -0.17(-0.43-0.10) |
|  | 70-85*stage 3 | 1.22(0.31-2.13) | 1.21(0.35-2.06) | 0.32(-0.08-0.71) | 0.32(-0.06-0.69) |

^a^ Unadjusted; ^b^ Adjusted for smoke, drink, diabetes, body mass index, family history of diabetes, family history of stroke, family history of cardiovascular disease, family history of hypertension.
